# Usability vulnerabilities of elderly adults in at-home COVID-19 self-test kits: findings from a South Korea usability study

**DOI:** 10.1186/s12889-025-25798-z

**Published:** 2025-11-28

**Authors:** Hyeong-Guk Son, Jee-Won Moon, Chan-Jin Choi, Ji-Ae Kim, Song-Ee Kim, Jae-Woo Hong, Il-Ho Park

**Affiliations:** 1 Department of Medical Device Usability Test Center, Guro Hospital, Korea University College of Medicine, 80, Guro-dong, Guro-gu, Seoul, 152-703 South Korea; 2 Department of Otorhinolaryngology–Head and Neck Surgery, Guro Hospital, University College of Medicine, 80, Guro-dong, Guro-gu, Seoul, 152-703 South Korea

**Keywords:** COVID-19 self-test kit, Usability, Human error analysis, Home health, Medical devices

## Abstract

**Background:**

The COVID-19 pandemic led to the widespread adoption of at-home COVID-19 self-test kits as accessible alternatives to clinical diagnostics. However, laypersons may have a limited understanding of use-related risk when operating these devices. Identifying how demographic factors such as sex, education level, and age influence use errors can guide improvements in the usability and safety of home-based medical devices.

**Methods:**

We recorded the frequency of use errors for each participant, along with their self-rated task understanding and satisfaction on a 5-point scale. We analyzed this data to compare error rates across different age, sex, and education groups. Furthermore, we used statistical models to determine the influence of these demographic factors on the likelihood of error occurrence and the total number of errors. Self-rated task understanding and satisfaction scores were also compared between participants who did and did not commit errors.

**Results:**

A total of 52 participants (17.2%) committed at least one use error, resulting in 123 errors recorded in total. Error rates increased markedly with age, ranging from 0% among adolescents (14–19 years), 7.25% in participants in their 20s, 5.08% in their 30s, 16.67% in their 40s, 30.77% in their 50s, 46.15% in their 60s, to 100% in participants in their 70s. Each additional year of age was associated with a 9.5% increase in the odds of any use error (OR = 1.095, 95% CI 1.064–1.127; *p* < 0.001) and a 7.9% increase in error counts (IRR = 1.079, 95% CI 1.053–1.106; *p* < 0.001) in pooled models adjusted for kit and calendar time. Tasks involving buffer dispensing and sample mixing were the most error-prone. Participants who committed errors reported significantly lower self-rated task understanding and satisfaction, with the effect especially pronounced among those aged 60 and above.

**Conclusions:**

Age significantly affected the usability of COVID-19 self-test kits, with older adults being more prone to errors, likely due to cognitive misunderstandings rather than physical limitations. Simplifying task steps and improving interface clarity, when tailored to older users’ cognitive needs, are crucial for safer use. These findings suggest that age-inclusive design improvements and clearer instructions can support safer and more effective self-testing for users of all ages.

**Supplementary Information:**

The online version contains supplementary material available at 10.1186/s12889-025-25798-z.

## Introduction

 The Coronavirus disease 2019 (COVID-19) pandemic, an unprecedented global crisis, has severely strained healthcare systems and heightened the demand for rapid and accessible diagnostic solutions [1–3]. One notable advancement in the pandemic response has been the increased availability of at-home COVID-19 self-test kits, which allow individuals to self-diagnose and manage potential infections outside of traditional clinical settings [2, 4–6]. Initially, the public expressed concerns about the proficiency required to perform nasopharyngeal swab-based self-tests. This sampling procedure had historically been performed only by trained medical professionals [3, 4]. To address these concerns, the sampling method was modified to use a less-invasive nasal swab approach [3, 4, 7]. However, the nature and extent of the use error that laypersons encounter when operating these kits remain poorly understood [4, 7, 8].

To ensure the safe use of COVID-19 self-test kits, the U.S. Food and Drug Administration (FDA) mandated usability testing involving at least 100 laypersons (a notable increase from the standard 15 participants), all with no prior self-testing experience. Based on these evaluations, the FDA approved the first COVID-19 self-test kits on November 17, 2020 [9–11]. The guidelines were later revised to recommend two types of usability testing: one with at least 15 laypersons using the kit independently and another with at least 15 laypersons using it under supervision [3, 12].

Although these kits became widely available and thus more familiar to the public during the pandemic, publicly available data on the use-related risk faced by inexperienced users remain limited [7, 13, 14]. While anecdotal and observational reports have accumulated, few studies have systematically analyzed how first-time users interact with self-test kits and which specific tasks or user characteristics contribute [5, 8, 14, 15]. Understanding such patterns is essential for identifying vulnerable user groups.

This study aimed to determine whether demographic characteristics—specifically age, sex, and education level—are associated with the likelihood and frequency of use errors during first-time COVID-19 self-testing. Although many factors (such as device design, training, and environment) can influence use errors, our analysis focused specifically on sex, education level, and age as potential determinants [3, 4] [7, 8, 14, 16]. Cognitive factors such as health literacy and memory load were beyond the scope of this study.

Our findings provide empirical evidence on demographic determinants of use errors. This evidence can support future human factors research and inform the design of safer, more accessible self-testing devices for diverse populations.

## Materials and methods

### Overview of usability testing design

Usability testing was conducted between 2021 and 2022 at the Korea University Guro Hospital Usability Test Center (KUTC), which was established with support from the South Korean Ministry of Health and Welfare. The tests were performed on three nasal swab-based COVID-19 self-test kits prior to their commercial approval, in accordance with the Korean Ministry of Food and Drug Safety (MFDS) guidelines and aligned with FDA usability standards [3]. Each test involved more than 100 laypersons with no prior self-testing experience.

### Usability testing environment

Usability testing was conducted in accordance with IEC 62366-1:2015 + AMD1:2020 and IEC TR 62366-2:2016, which provide frameworks for identifying and mitigating use-related risk [4, 17, 18]. The testing environment simulated manufacturer-defined home-use conditions, including a noise level of 40–50 dB, lighting of 300–500 lx, a temperature of 15–30 °C, and humidity of 30%–50%. For the usability test, we set up an independent observation and testing room according to Human Factors Engineering guidelines (ANSI/AAMI HE75:2009/(R)2018). Participants and study personnel were placed in separate rooms to minimize interaction. All test sessions were recorded using a video camera (Axis P5624, Sweden) and an audio system (Axis P8221, Sweden) installed in the test room.

### Participant recruitment criteria

Participant enrollment numbers followed Korean MFDS guidelines updated in March and August 2021. This study was approved by the Institutional Review Board of Korea University Guro Hospital (approval Nos. 2021GR0229, 2021GR0317, 2021GR0600). Participants were recruited through open calls via public advertisements posted at the hospital, on subway bulletin boards, and through online banner ads. Participants were stratified by age (per each manufacturer’s specifications) with no restrictions on sex or education level, to ensure a diverse sample. Only individuals with no prior experience using personal in vitro diagnostic devices were recruited. All participants provided informed consent and could withdraw from the study at any time. One participant voluntarily withdrew due to a misunderstanding of the study’s purpose.

### Study procedure

Upon arrival at the study site, participants received a detailed explanation of the study and provided written informed consent. They were then asked to perform a series of predefined tasks according to the usability test protocol. The detailed procedural flow for each participant, from initial enrollment to final task completion, is summarized in Supplementary Table S1-S2.

### Demographic distribution of participants

Three separate usability studies were conducted, one for each of the three COVID-19 self-test kits. Each kit’s manufacturer defined its intended user age range according to MFDS guidelines and set specific age groups for participant recruitment reflecting Korean population demographics [2]. All three products included participants aged 20–64 years. Additionally, one product included adolescents (14–19 years), and another included participants aged 65 and above (Table 1). Educational level was categorized into two groups: high school education or below, and college education or above. A high school diploma was regarded as the baseline education level because secondary education is mandatory in Korea.

**Table 1:** Intended user age range and participant recruitment criteria as defined by the manufacturer

Test	Intended user age range	Age group (years)	Number of Participants (*n*)
Test A	18–79	18–37	40
		48–57	40
		58–79	21
Test B	14–65	14–18	25
		19–29	25
		30–49	27
		50–65	25
Test C	19–64	19–29	25
		30–39	25
		40–49	25
		50–64	25

### Definition and classification of use errors

Use errors were defined according to IEC 62366-1:2015 + AMD1:2020 as any action or inaction deviating from the intended use that results in an unintended outcome (e.g., During specimen collection, the swab was advanced beyond the prescribed insertion depth within the nasal cavity.)

Situations in which a use error was narrowly avoided without external intervention were classified as “close calls.” Instances of confusion or hesitation that did not result in an actual error were categorized as “difficulties.” In Test A, we observed one close call (related to the specimen collection method) and two difficulties (one with securing the tube and another with performing the specimen collection). In Test C, one use difficulty occurred during the interpretation of a faint positive result. Due to the low frequency of these events and their limited analytical reliability, close calls and use difficulties were excluded from the final analysis.

All usability tests were video and audio recorded for detailed analysis. During each live session, a test manager and an observer jointly documented any observed deviations from the intended use. To ensure consistent application of the error criteria across all 303 participants and three test kits, the final classification of all potential use errors was conducted by a single, experienced coder (the test manager, with over five years of experience in usability engineering). The classified errors were subsequently documented in a formal test report, which was then reviewed and confirmed by the head of the usability test center (KUTC). This entire process is part of our center’s standard operating procedure, which is certified under the ISO 13485:2016 quality management system for medical devices.

### Post-task satisfaction assessment

After each task, participants rated how well they understood the product on a 5-point Likert scale and provided a brief verbal explanation for their rating. These understanding/satisfaction scores were collected for all task scenarios and later grouped by participant sex, education level, and age group for analysis.

### Configuration and use scenarios of COVID-19 self-testing kits

**Fig. 1:**
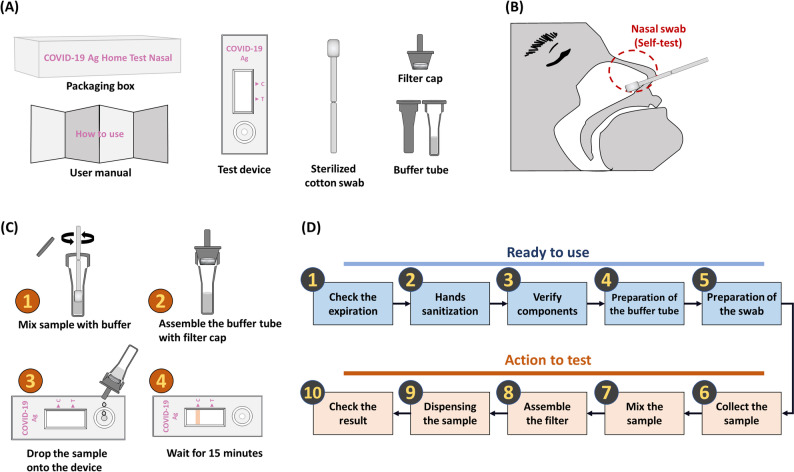
Overview of COVID-19 Self-Testing Kit. (**A**) Kit components. (**B**) Nasal swab collection position. (**C**) Simplified instructions. (**D**) Classification of task scenarios used for error analysis

We analyzed data from three self-testing kits evaluated between 2021 and 2022. Although the kits differed in packaging, swab length, design, and manual wording, they shared the same core components: packaging, a user manual, a test device, a sterilized cotton swab, a filter cap, and a buffer tube (Fig. 1A). The user manual for each kit was provided as a paper leaflet containing both text and pictograms. To simulate a realistic home-use scenario, participants were allowed to consult the manual at any time during the test. All kits followed the same procedure: an anterior nasal swab for sample collection, application of the sample to the test device using the buffer tube, and then result verification (Fig. 1B and C). The usability tests followed scenarios derived from each manufacturer’s risk management documentation, including Use Failure Mode and Effects Analysis (UFMEA). While the detailed number of steps per kit ranged from 21 to 33, the use procedures were divided into two phases—“ready-to-use” and “action-to-test”—with five key tasks in each phase for comparative analysis (Fig. 1D). The detailed classification of the original steps into this 10-task framework is provided in Supplementary Table S3. This standardized 10-task framework allowed comparison across kits, even though the exact step counts differed by product. Although the three kits differed slightly in packaging layout, swab length, and manual phrasing, all followed a comparable functional process and included the same core components. These minor design differences among the three self-test kits are summarized in Table 2.

**Table 2:** Structural and instructional characteristics of the three COVID-19 self-test kits evaluated

Feature	Test A	Test B	Test C
Package box	Outer and inner packaging boxes	Outer and inner packaging boxes	Outer packaging box only
User manual	Folded A4-size leaflet; differences observed in content and layout	Folded A4-size leaflet; differences observed in content and layout	Folded A4-size leaflet; differences observed in content and layout
Test device	Substantially equivalent design across all three kits	Substantially equivalent design	Substantially equivalent design
Cotton swab length	*150 mm	150 mm	100 mm
	*Only Test A, the swab was broken after use and inserted into the buffer tube for extraction.		
Buffer tube	Substantially equivalent design	Substantially equivalent design	Substantially equivalent design
Filter cap	Substantially equivalent design	Substantially equivalent design	Substantially equivalent design
Buffer tube holder location	Mounted upright in a plastic tray inside the inner box	Mounted upright in a plastic tray inside the inner box	Mounted in a cardboard holder inside the outer packaging box

### Statistical analysis

We conducted all analyses in R (version 4.5.1; R Foundation for Statistical Computing, Vienna, Austria). Descriptive statistics summarized demographics, use-error frequencies, and satisfaction scores. Proportions of participants with ≥ 1 use error were compared across sex, education, and age using chi-squared or Fisher’s exact tests as appropriate; for comparisons spanning > 2 age groups, we used chi-squared tests with Monte Carlo simulation (10,000 replicates) to address small expected counts. We fit pooled regression models with fixed effects for test kit (A/B/C) and a continuous calendar-time covariate (days since June 1, 2021). For the binary outcome (any use error) we used logistic regression; for error counts we used negative binomial models when the Poisson dispersion ratio exceeded ~ 1.2, otherwise Poisson. In the primary pooled model, the Poisson dispersion ratio was 3.66, supporting the negative binomial specification. Zero inflation was evaluated with DHARMa residual diagnostics and was not indicated (*p* = 0.964); therefore, a standard negative binomial model was retained. Sensitivity analyses included mixed-effects models with a random intercept for kit and models with cluster-robust standard errors by kit. Primary multivariable regressions were considered confirmatory for our hypotheses regarding demographic predictors; all other subgroup comparisons (e.g., across tasks or age groups) were exploratory without multiplicity adjustment. For satisfaction scores, we used non-parametric tests: Kruskal–Wallis for multiple groups and Mann–Whitney U for two-group comparisons. All tests were two-sided with α = 0.05, and exact p-values are reported to three decimals.

## Results

Table 3 summarizes the demographic characteristics of the 303 participants across the three usability tests. Participants were recruited according to each kit manufacturer’s specified user age range, yielding sample sizes of 101 in Test A, 102 in Test B, and 100 in Test C. Overall, female participants (193, 63.7%) outnumbered male participants (110, 36.3%). Educational attainment was similarly skewed: 66.7% of participants (202 of 303) had a college degree or higher, whereas 33.3% (101 of 303) had a high school education or below.

**Table 3:** Demographic distribution of participants by age, sex, and education level

	Test A	Test B	Test C	Total
Total *N*	101	102	100	303
**Age (years), N**				
14–19	N/A	27	N/A	27
20–29	21	23	25	69
30–39	23	11	25	59
40–49	25	16	25	66
50–59	13	19	20	52
60–69	15	6	5	26
70–79	4	N/A	N/A	4
**Sex**,** N**				
Male	41	32	37	110
Female	60	70	63	193
**Education**,** N**				
High school education or below	22	54	25	101
College degree or above	79	48	75	202

Table 4 shows the distribution of use errors across the different demographic subgroups. In total, 52 participants (17.16%) experienced at least one use error, resulting in 123 errors recorded in total. The highest proportion of participants with use errors was observed in Test C (21.00%), followed by Test A (19.80%) and Test B (10.78%). Notably, Test A accounted for the most errors (75, over 60% of all errors) despite similar sample sizes across tests.

**Table 4:** Distribution of use errors by demographic characteristics

	Test A	Test B	Test C	Total
**Total N**	101	102	100	303
Correct use (%, n/N)	80.20% (81/101)	89.22% (91/102)	79.00% (79/100)	82.84% (251/303)
Use errors (%, n/N)	19.80% (20/101)	10.78% (11/102)	21.00% (21/100)	17.16% (52/303)
Number of use errors	75	16	32	123
**Use errors by age (%**,** n/N)**				
14–19	N/A	0% (0/27)	N/A	0% (0/27)
20–29	9.52% (2/21)	4.35% (1/23)	8.00% (2/25)	7.25% (5/69)
30–39	0% (0/23)	9.09% (1/11)	8.00% (2/25)	5.08% (3/59)
40–49	8.00% (2/25)	12.50% (2/16)	28.00% (7/25)	16.67% (11/66)
50–59	38.46% (5/13)	21.05% (4/19)	35.00% (7/20)	30.77% (16/52)
60–69	46.67% (7/15)	50.00% (3/6)	40.00% (2/5)	46.15% (12/26)
70–79	100% (4/4)	N/A	N/A	100% (4/4)
**Use errors by Sex (%**,** n/N)**				
Male	9.76% (4/41)	9.38% (3/32)	27.03% (10/37)	15.45% (17/110)
Female	26.67% (16/60)	11.43% (8/70)	15.87% (10/63)	17.62% (34/193)
**Use errors by Education (%**,** n/N)**				
High school education or below	36.36% (8/22)	9.26% (5/54)	28.00% (7/25)	19.80% (20/101)
College degree or above	15.19% (12/79)	12.50% (6/48)	17.33% (13/75)	15.35% (31/202)

### Influence of sex and educational level on use errors

**Fig. 2:**
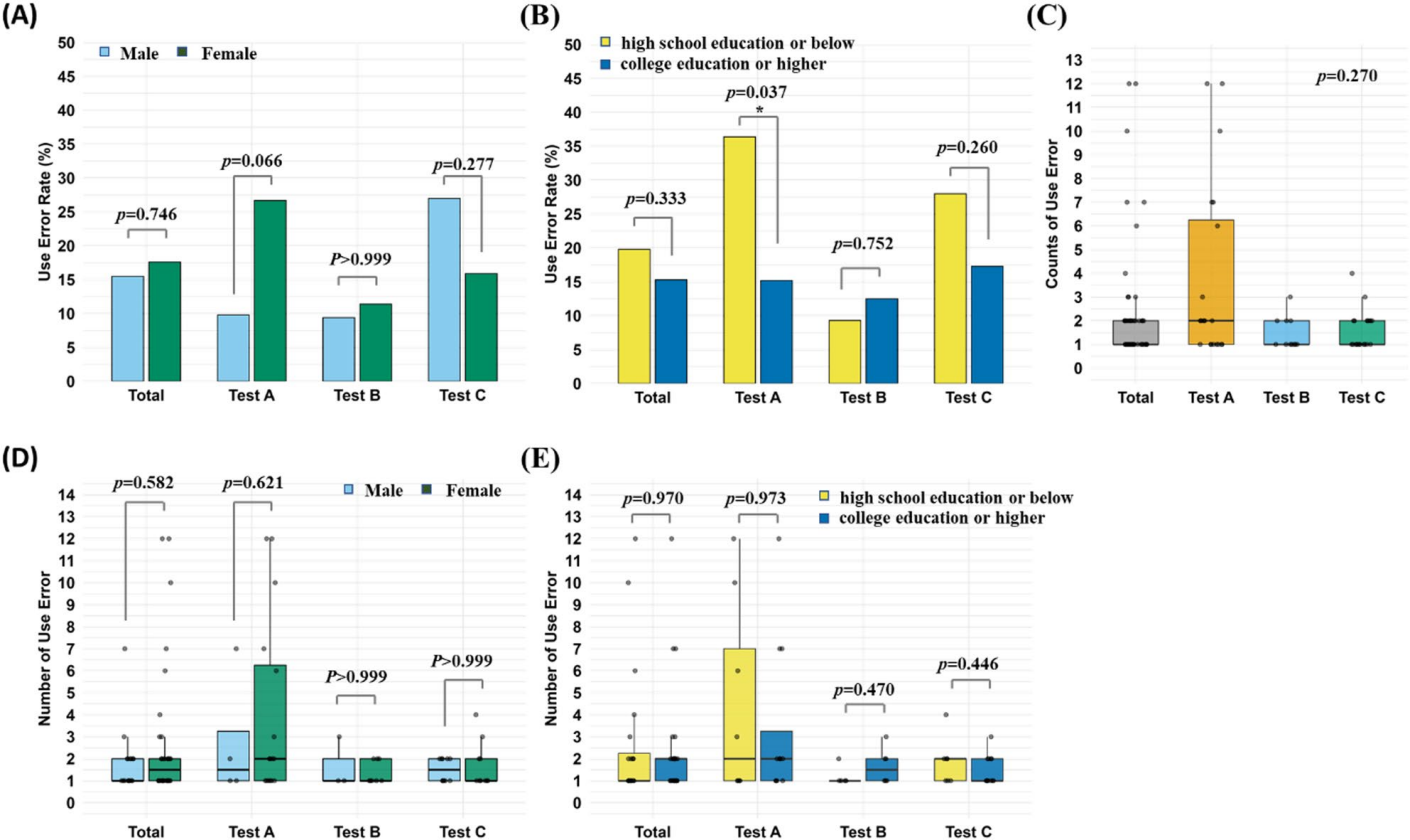
Use error rates and counts by sex and education level across the three self-test kits. Percentages of participants with ≥ 1 use error (**A**) by sex and (**B**) by education level, respectively. (**C**) Distribution of use error counts per participant among those who made errors. Total number of use errors per participant (**D**) by sex and (**E**) by education level, respectively. Chi-square tests compared error rates by sex; Fisher’s exact test was used for error rates by education level (due to low expected counts). Mann–Whitney U tests compared total error counts by sex and by education level

We assessed whether sex and education level influenced both the likelihood of a use error and the number of errors per person. In the total sample (*N* = 303), 17.16% of participants (52 individuals) experienced at least one use error. The error rate was 15.45% for males (17/110) and 17.62% for females (34/193). This slight female–male difference was not statistically significant (Fig. 2A). Sex-based error rates varied by test: in Test A, females had a higher error rate than males (26.67% vs. 9.76%), whereas in Test C, males had a higher error rate than females (27.03% vs. 15.87%). Test B showed virtually no sex-related difference in error rates. However, none of these differences was statistically significant (all *p* > 0.05).

Educational level showed a similar trend: participants with a high school education or lower had a slightly higher error rate than those with a college degree or above (19.80% vs. 15.35%), but this difference was not statistically significant (Fig. 2B). A significant difference was observed only in Test A, where the lower-education group had an error rate of 36.36% compared to 15.19% in the higher-education group (*p* < 0.05). No significant educational differences were found in Tests B or C.

Among the 52 participants who committed errors, a total of 123 errors were recorded. Of those who erred, the majority (28 participants) made only one error; 14 made two errors, and 10 made three or more (Fig. 2C). Test A had the highest error frequency, with error-prone participants averaging 3.75 errors each (SD = 3.82, Max = 12) compared to averages of 1.45 (SD = 0.69) in Test B and 1.60 (SD = 0.82) in Test C.

Analysis by sex revealed that females had a higher average number of use errors per person than males (2.71 vs. 1.82 among participants who made at least one error in the total sample). This sex difference was most pronounced in Test A (female error-makers averaged 4.00 errors vs. 2.75 for male error-makers), whereas Tests B and C showed only minor differences. However, none of these comparisons was statistically significant (all *p* > 0.05; Fig. 2D).

Stratifying by education level revealed a similar pattern: in Test A, participants with lower education had a slightly higher mean error count than those with higher education (4.38 vs. 3.33), and similarly in Test C (1.86 vs. 1.46), whereas in Test B the higher-educated group had the higher mean count. Again, these differences were not statistically significant (Fig. 2E).

Overall, neither sex nor education level emerged as significant determinants of use error occurrence or frequency, although greater variability was observed among the lower-education group in some cases (e.g., Test A).

### Influence of age on use errors

**Fig. 3:**
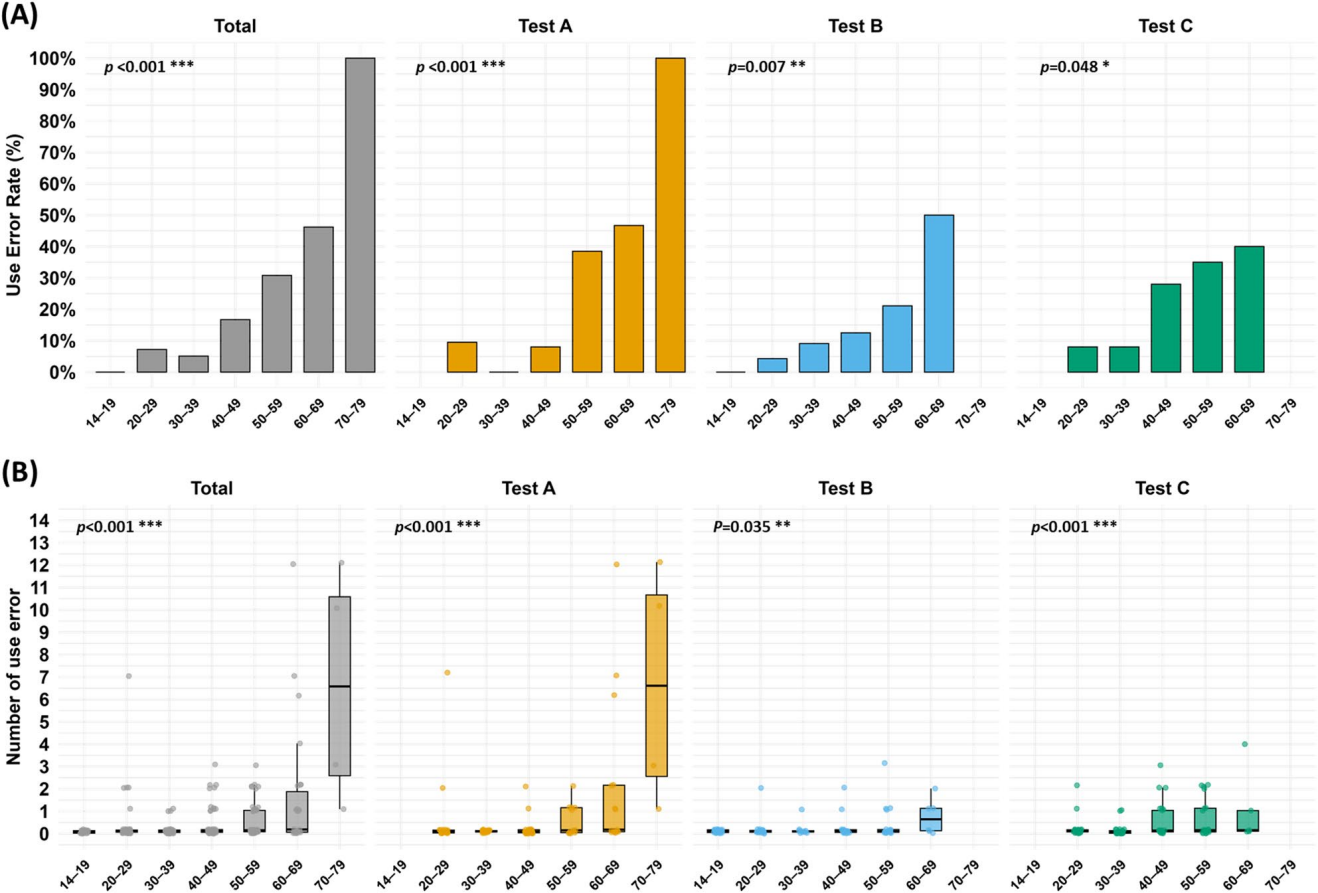
Age-related trends in use errors. (**A**) Percentage of participants with ≥ 1 use error by age group (10-year intervals) for the total sample and each test (Test A, Test B, Test C). Chi-squared tests assessed differences in proportions by age. (**B**) Distribution of use error counts per participant by age group, for each test group and the total sample. Poisson regression models were fitted to assess whether error frequency differed significantly across age groups

Use error rates increased progressively with age in the total sample (Fig. 3A). No errors were observed among adolescents aged 14–19 (this age group was included only in Test B). In contrast, error rates climbed from 7.25% in the 20–29 age group to 46.15% in the 60–69 group. Moreover, every participant in the 70–79 age group (4/4 participants) experienced at least one error (100%). However, given the very small sample size in the 70–79 group, this 100% error rate should be interpreted with caution. A chi-squared test with Monte Carlo simulation confirmed a significant association between age group and use error occurrence (χ² = 58.76, *p* < 0.001).

In each individual test, the age-related trend persisted. For example, in Test A, use error rates increased from 0% in participants in their 10 s to 30.77% in those 50–59, and to 100% in those 70–79. Similar age-related patterns were observed in Tests B and C as well. Chi-squared tests within each test confirmed significant age associations in Test A (χ² = 35.14, *p* < 0.001), Test B (χ² = 16.01, *p* = 0.007), and Test C (χ² = 9.56, *p* = 0.048).

Figure 3B also illustrates the total number of use errors per age group. When pooling all tests, older participants contributed more errors overall, peaking at 16 errors in the 50–59 group and 12 errors in the 60–69 group, whereas the youngest group (14–19) had none. The youngest (14–19) and the 30–39 age groups had the fewest errors (0 and 3 total errors, respectively). In Test A, the highest total errors were recorded in the 60–69 (7 errors) and 70–79 (4 errors) age groups, while no errors occurred among those aged 30–39. In Test B, total error count increased with age, reaching 4 errors in the 50–59 group and 3 errors in the 60–69 group. In Test C, the 40–49 and 50–59 age groups had the highest error totals (7 errors each), while the 20–29, 30–39, and 60–69 groups each had 2 errors.

To further examine the relationship between age and use error frequency, we fit Poisson regression models with age group as a categorical predictor. These models indicated significant associations in all cases: overall (*p* < 0.001), Test A (*p* < 0.001), Test B (*p* = 0.004), and Test C (*p* < 0.001). These findings reinforce that age is a strong predictor of use error frequency, with older adults consistently exhibiting higher error rates in all test settings.

### Mixed-effects regression strengthens evidence for age-related use errors

To evaluate age, sex, and education simultaneously while accounting for between-kit heterogeneity and period effects, we fit mixed-effects models (*N* = 303): a mixed-effects logistic model for any use error and a mixed-effects negative binomial model for the error count. Both models included a random intercept for test kit and were adjusted for calendar time (days since 2021-06-01). Model family for the count outcome was chosen per dispersion diagnostics (see Supplementary Table S4).

Age remained the only significant predictor in both models. In the logistic model, each additional year of age was associated with a 9.5% increase in the odds of having ≥ 1 use error (OR = 1.095, 95% CI: 1.064–1.127; *p* < 0.001). In the negative binomial model, the expected error count increased by 7.9% per year (IRR = 1.079, 95% CI: 1.053–1.106; *p* < 0.001). Neither sex nor education showed statistically significant associations. Male vs. female yielded OR = 1.731 (95% CI: 0.811–3.692; *p* = 0.156) and IRR = 1.530 (95% CI: 0.733–3.189; *p* = 0.257). ≤ High school vs. ≥ College yielded OR = 1.429 (95% CI: 0.691–2.959; *p* = 0.336) and IRR = 1.218 (95% CI: 0.606–2.445; *p* = 0.580). These findings are consistent with Table 5 and robust to model specification; full coefficients are reported in Supplementary Table S5.

**Table 5:** Mixed-effects regression analyses predicting the likelihood (logistic) and frequency (negative binomial) of use errors (*N* = 303)

Variable	OR (95% CI)	*p*-value	IRR (95% CI)	*p*-value
Age (per 1 year increase)	1.095 (1.064–1.127)	< 0.001 ***	1.079 (1.053–1.106)	< 0.001 ***
Sex (Male vs. Female)	1.731 (0.811–3.692)	0.156	1.530 (0.733–3.189)	0.257
Education (≤ High school vs. ≥ College)	1.429 (0.691–2.959)	0.336	1.218 (0.606–2.445)	0.580
Model specification: mixed-effects logistic (any use error) and mixed-effects negative binomial (error count), random intercept for test kit, adjusted for calendar time (days since 2021-06-01)				
Exact two-sided *p*-values are reported to three decimals; values < 0.001 are shown as “<0.001”				

*OR* Odds ratio, *IRR* Incidence rate ratio, *CI* Confidence interval

### Task scenarios and use error occurrence

**Fig. 4:**
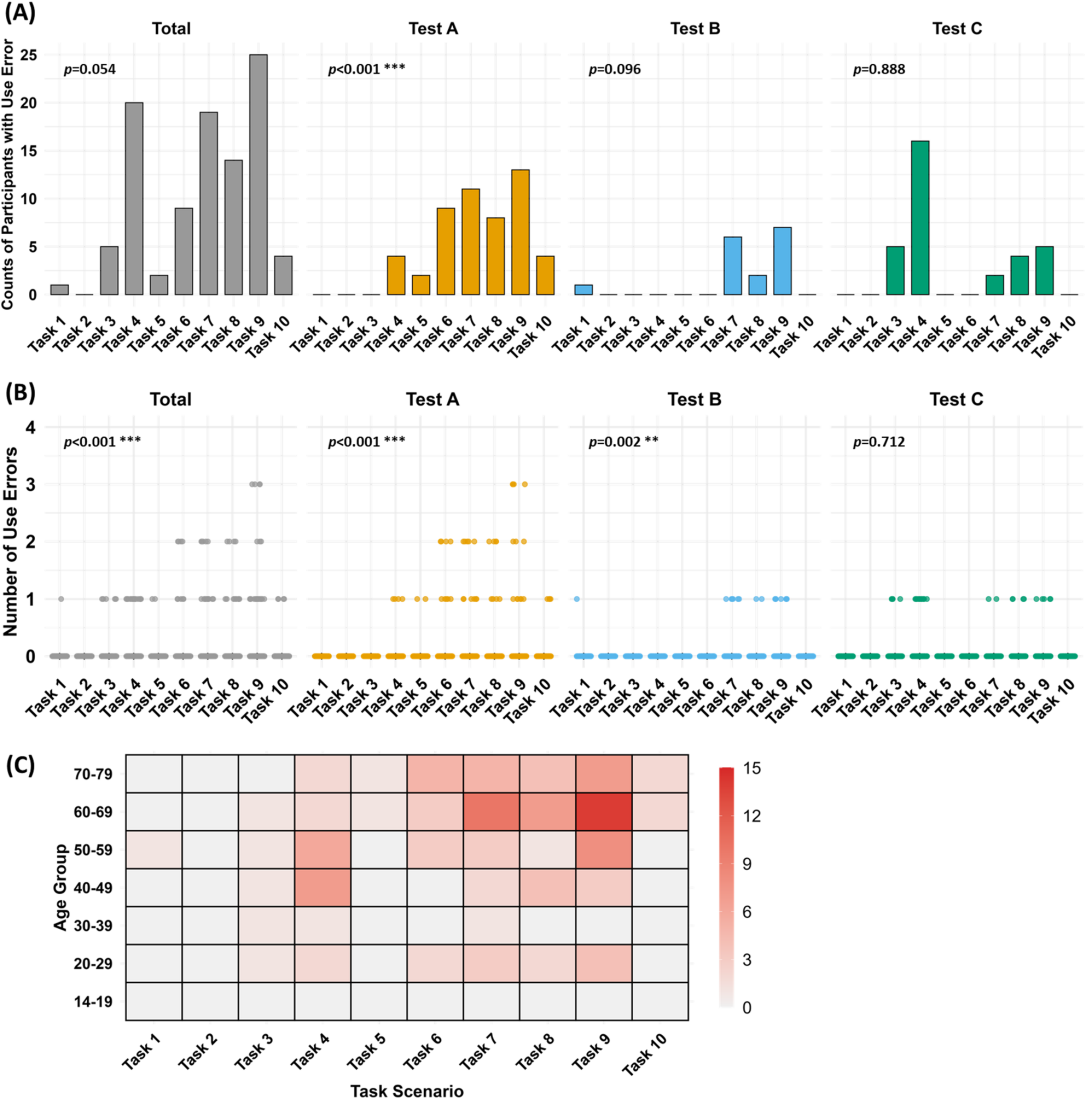
Distribution of use errors by task scenarios. (**A**) Number of participants who experienced ≥ 1 use error in each task scenario, shown for the total sample and by test group (A, B, C). (**B**) Number of use errors per participant for each task scenario, stratified by test group. (**C**) Heatmap of total use errors per task scenario across age groups

To identify which task scenarios were most error-prone, we analyzed the number of participants who committed at least one use error in each task scenario (Fig. 4A). Table 6 summarizes each task scenario along with the corresponding typical use errors observed during testing. Task 9 (buffer dispensing) had the most participants with errors (25 participants), followed by Task 4 (20 participants), Task 7 (19 participants), and Task 8 (14 participants).

**Table 6:** Task Scenarios, intended uses, and observed use errors in At-Home COVID-19 Self-Testing

Task Scenarios	Intended use	Typical use errors
Task 1 Check the expiration	Inspect the product for physical damage and verify the expiration date.	Failure to verify the product prior to use
Task 2 Hand sanitization	Wash and dry hands	Failure to perform hand sanitization procedures
Task 3 Verify components	Confirm the presence of all components and recognize their respective functions.	Inability to identify the names and functions of kit components Did not recognize that the buffer solution was prefilled in the tube
Task 4 Preparation of the buffer tube	Remove the packaging from the buffer tube and buffer cap.	Difficulty identifying the buffer tube and cap, leading to attempting specimen collection without preparing them
Task 5 Preparation of the swab	Remove the swab packaging and review the user manual for sample collection.	Failure to secure the buffer tube, resulting in spillage of buffer solution. Swab was immersed in the buffer before collecting the nasal specimen
Task 6 Collect the sample	Collect a nasal sample using the swab	Collected the specimen deeper than intended or only from one nostril
Task 7 Mix the sample	Mix the collected sample thoroughly with the buffer solution	Insufficient mixing of the specimen with the buffer solution. Failure to recognize that the swab should be broken at the marked breakpoint after sufficient mixing
Task 8 Assemble the filter	Correctly attach the filter and filter cap in the appropriate orientation	Did not recognize the correct orientation for attaching the filter cap; failure to assemble the filter cap
Task 9-Dispensing the buffer	Dispense 2–3 drops of the mixed buffer solution into the sample well of the test kit	Difficulty identifying the correct drop location Drop capacity differs from the number of drops intended by the manufacturer
Task 10-Check the results	Interpret the test result as invalid, negative, weak positive, or positive	Misinterpretation of faint positive results or failure to understand result interpretation correctly

In test-specific analyses, the error-prone tasks differed somewhat between kits: Test A had the most errors in Task 7 (11 participants) and Task 9 (13 participants); Test B had the most in Task 9 (7) and Task 7 (6); Test C had the most in Task 4 (16) followed by Task 3 (5). To test for differences in the number of error-prone participants across tasks, we used a negative binomial regression model for the combined dataset (dispersion > 1.2 indicating overdispersion). Only Test A showed a statistically significant variation in error occurrence across tasks (*p* < 0.001). The overall sample showed a marginally non-significant trend (*p* = 0.054), and no significant task effect was found in Test B (*p* = 0.096) or Test C (*p* = 0.888).

Next, we analyzed the number of use errors per participant for each task (Fig. 4B). Participants committed up to three errors in a single task; notably, instances of repeated errors (two or more in one task) were observed only in Test A. In Tests B and C, each participant had at most one error per task. Negative binomial regression confirmed a significant effect of task scenario on error counts in the overall sample (dispersion = 1.24, *p* < 0.001) and in Test A (dispersion = 1.29, *p* < 0.001). In Test B, where dispersion was low (0.95), a Poisson regression indicated a significant association (*p* = 0.002). No significant task-related effect was found in Test C (dispersion = 0.97; *p* = 0.712).

To explore age-related patterns, we generated a heatmap (Fig. 4C) to visualize task-specific error distributions across age groups. Older age groups (60–69 and 70–79 years) consistently showed higher error counts across multiple tasks. For instance, in the 60–69 age group, Task 9 had the highest error count (14), followed by Task 7 (10) and Task 8 (7). Similarly, in the 70–79 group, errors were most frequent in Task 9 (7 errors), Task 6 (5 errors), and Task 7 (5 errors). Participants in the 40–59 year range showed a moderate number of errors, especially in Task 4 and Task 9. To quantify these trends, we calculated Spearman rank correlation coefficients between participant age and the number of use errors per task. Positive correlations were found for multiple tasks, with Task 9 being the highest (ρ = 0.320), followed by Task 7 (ρ = 0.258), Task 6 (ρ = 0.207), and Task 4 (ρ = 0.196).

### Analysis of self-rated task understanding and satisfaction scores

**Fig. 5:**
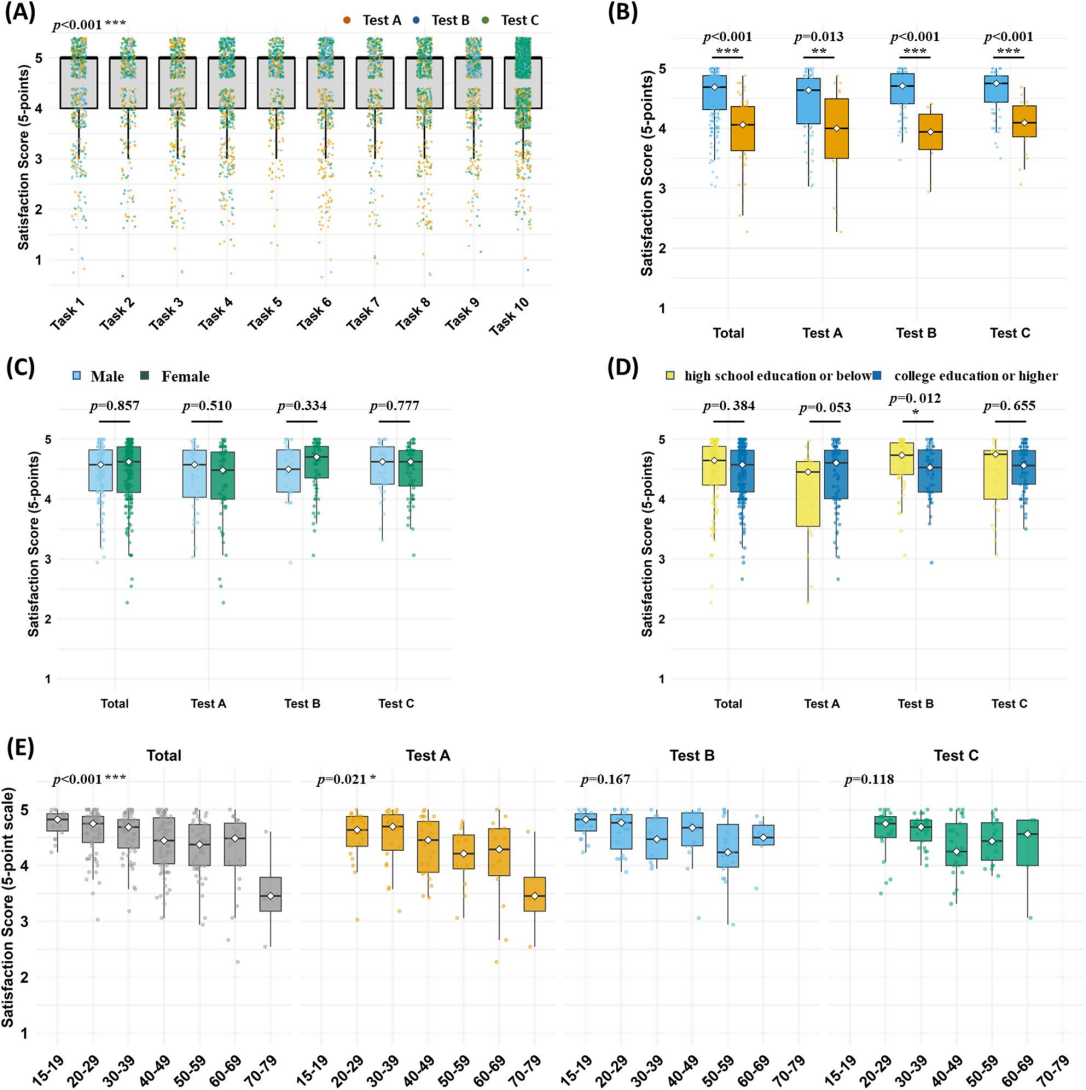
Scores for self-rated task understanding and satisfaction across tasks and participant characteristics. (**A**) Box plots of satisfaction scores for tasks 1–10 (5-point Likert scale). (**B**) Satisfaction scores for participants with no use errors vs. those with ≥ 1 error, shown for the total sample and each test group. (**C**) Satisfaction scores by sex. (**D**) Satisfaction scores by education level (high school or below vs. college or above). (**E**) Satisfaction scores by age group (10-year intervals) in each test. Boxes represent interquartile range; horizontal line = median; points = individual participants

Participants rated their task understanding and satisfaction after each task on a 5-point Likert scale (Methods; Fig. 5A). Because ratings were strongly right-skewed with ceiling effects (medians = 5; IQR 4–5), we report mean ± SD to capture subtle between-task differences while preserving the original scale. Across tasks 1–10, mean scores were uniformly high (4.35–4.62). The highest mean scores were for Task 5 (mean ± SD: 4.58 ± 0.71) and Task 10 (4.62 ± 0.66), whereas Tasks 3, 7, and 8 had the lowest scores (around 4.35 ± 0.88 for each). A Kruskal–Wallis test indicated a statistically significant difference in satisfaction across tasks (*p* < 0.001).

We then examined satisfaction differences between participants who had no errors (Correct Use group) and those who had at least one error (Use Error group). In the overall sample, participants with no errors reported significantly higher satisfaction (mean ± SD: 4.52 ± 0.47) than those with errors (4.23 ± 0.53; *p* < 0.001, Mann–Whitney U) (Fig. 5B). The same pattern was observed within each individual test. For Test A, the no-error group scored higher (4.40 ± 0.55) than the error group (4.09 ± 0.68; *p* < 0.001). Similarly, in Test B (4.56 ± 0.43 vs. 4.29 ± 0.53, *p* < 0.001) and Test C (4.55 ± 0.40 vs. 4.26 ± 0.44, *p* < 0.001), satisfaction was significantly higher for participants without errors. Sex-based comparisons revealed no significant difference in satisfaction scores. In the overall sample, males and females both reported essentially the same mean satisfaction (~ 4.45, no significant difference), and no statistically significant differences were observed between sexes in any test group (Fig. 5C).

Educational level was not significantly associated with satisfaction in the total sample (*p* = 0.384). Participants with a high school education or below reported a mean satisfaction score of 4.44 ± 0.59 (mean ± SD), while those with a college degree or higher scored 4.45 ± 0.46 (Fig. 5D), indicating virtually no difference. Subgroup analyses, however, revealed a statistically significant difference in Test B (*p* = 0.012): participants with a college education or higher reported lower satisfaction (4.44 ± 0.44) compared to those with a high school education or below (4.61 ± 0.43). This finding was not observed in Test A (*p* = 0.053) or Test C (*p* = 0.655), where differences between education groups were not statistically significant.

To examine age differences in satisfaction, we stratified participants into 10-year age groups. In the overall sample, satisfaction scores differed significantly by age group (Kruskal–Wallis, *p* < 0.001; Fig. 5E). Participants in their 20 s and 30 s reported higher satisfaction compared to participants aged 60 and above. Subgroup analyses showed that this age effect was significant only in Test A; no statistically significant differences were observed in Test B or Test C.

## Discussion

This study found that age was the dominant factor influencing the usability of at-home COVID-19 self-test kits, whereas sex and education level had no significant impact after accounting for age. Older adults were far more prone to use errors than younger participants, even in a relatively healthy community sample. For instance, while virtually no adolescents or young adults committed errors, a majority of participants in their 60 s and all participants in their 70 s experienced at least one error (notwithstanding the small sample size in the oldest group). Each additional year of age increased the odds of an error by nearly 10% (*p* < 0.001), underscoring a strong age effect. This finding is consistent with gerontological research showing that normal cognitive aging—slower information processing, reduced working memory, and declines in executive function—can impair performance on complex tasks [19, 20]. This indicates that age-related cognitive changes likely overshadowed any minor usability differences attributable to sex or education in this context. The absence of sex and education effects in our results should be interpreted in light of this strong age influence, rather than as evidence that those factors never matter. It is also notable that our sample’s relatively homogeneous educational background (due to universal high school education in South Korea) may have made it harder to detect any subtle influence of education level [21, 22]. Overall, the age-related vulnerability in using these kits appears to far exceed any general demographic differences, suggesting that older adults have unique usability challenges with self-testing that younger users do not encounter to the same degree.

Notably, the nature of errors differed by age. Tasks requiring multiple steps and precise sequencing – in particular, sample mixing and buffer dispensing – were disproportionately error-prone among older participants. We observed that these errors generally did not stem from physical limitations (e.g., poor dexterity) but rather from cognitive misunderstandings. We observed that many older users became confused about the correct sequence or timing of steps and the interpretation of kit components, resulting in mistakes like skipping a step, assembling components in the wrong order, or misjudging the number of drops of buffer. Such errors indicate breakdowns in cognitive processing (memory, attention, or comprehension) rather than simple motor slips. This interpretation is supported by the fact that younger participants, who presumably have more robust working memory and faster information processing, rarely made these kinds of mistakes. In our study, younger users who did err typically made a single, isolated slip (for example, momentarily misreading an instruction), which they often caught or corrected, suggesting their errors were more due to general usability issues or momentary lapses. In contrast, older users tended to repeat errors or compound them, implying a deeper misunderstanding of the procedure that was not resolved during the self-test session. These observations underscore an important distinction between general usability problems and age-related vulnerabilities. Some usability issues (e.g., confusing instructions or ambiguous visual cues) can affect users of any age; indeed, even a few younger participants encountered minor confusion, indicating areas where the kit design could be universally improved. However, the persistent, systematic errors among older adults point to challenges that are specifically exacerbated by aging, such as difficulties with memory load and complex sequences [23–25]. This pattern resonates with human factors literature showing that as tasks become more complex or cognitively demanding, older adults are disproportionately affected due to normative declines in memory and attentional resources. In practical terms, the multi-step nature of the self-test, which involves preparing reagents, timing, and interpreting results, may impose a cognitive load that exceeds the comfortable capacity of many seniors, even if the same tasks are manageable for younger people. Prior usability studies have similarly found that older adults often struggle with procedural complexity and high mental workload when using novel medical or technological devices [24–26]. Our findings extend this knowledge by pinpointing specific self-test steps that overloaded older users’ cognitive abilities. For example, we suspect that some seniors overlooked critical information in the instructions or kit interface due to age-related limitations in attention and perception. In sum, the error patterns suggest that older adults’ challenges with the self-test kits were largely cognitive in nature: stemming from memory lapses, misunderstanding of sequences, and difficulty interpreting visual/textual information, rather than from lack of effort or gross motor impairment. Differentiating these age-driven errors from general design flaws is important because it indicates that simply training older users more may not fully solve the issue – instead, the design itself needs to account for age-related cognitive constraints [20, 23, 27]. The consequences of these cognitive challenges were evident not only in objective error rates but also in the subjective experience of older users. Participants aged 60 and above not only made more mistakes, but often made the same mistakes repeatedly within a single testing session. This persistence of errors suggests that once an older user formed an incorrect mental model of a step (for example, believing the test procedure was complete when it was not, or misidentifying a component’s purpose), it was difficult for them to adjust or recover. In essence, many older participants were likely operating at the limits of their working memory and comprehension capacity during the test, leaving little bandwidth to detect and correct errors on the fly. This interpretation is reinforced by the satisfaction ratings: although overall usability satisfaction was high across the sample (most users rated their experience quite positively), older adults consistently reported lower satisfaction and confidence in their understanding of the test. The drop in satisfaction among older users can be seen as a proxy for their increased frustration, uncertainty, and mental effort. From a human factors perspective, such lower self-reported satisfaction in older adults often correlates with higher perceived task difficulty and mental workload. The cognitive strain on older users likely not only caused more errors but also diminished their confidence in performing the test correctly. This has important public health implications: if older individuals doubt their ability to self-test accurately, they may avoid using these kits or perform them incorrectly without realizing it, potentially leading to false results and delayed care. Overall, these findings highlight that cognitive ergonomics – addressing how information is presented and how instructions align with users’ cognitive capabilities – is a critical aspect of safety for at-home medical testing, particularly for seniors. In line with principles from the *Handbook of Human Factors and Ergonomics* on designing for aging populations, our results underscore the value of simplifying tasks, providing clear cues, and reducing memory demands to accommodate older adults’ needs [27]. The observed age-related difficulties are not merely a user “learning” issue but reflect fundamental age-associated constraints; therefore, design solutions (and not just user training) are needed to bridge this gap [20].

Several limitations of this study should be noted when interpreting the results. First, the data were collected from a single country (South Korea) with a relatively homogeneous population in terms of educational background and cultural context. While this controlled some variables, it also means our findings might not generalize to settings where education levels vary more widely or where familiarity with self-testing technology is different. The lack of a significant education effect in our analysis could be partly due to the limited variability in education within our sample – in a more diverse population, education or health literacy might play a larger role in usability outcomes. Relatedly, we did not directly measure health literacy or cognitive ability of participants; we used formal education as a rough proxy, but this may not capture older adults’ true ability to comprehend medical instructions. Future research should include standardized health literacy assessments or cognitive screenings to see how these factors intersect with age in influencing errors. Second, Our qualitative observations suggest many errors by older adults were cognitive “mistakes” (due to misunderstanding) rather than motor “slips,” but without a formal error taxonomy or root-cause analysis we cannot quantify this precisely. Incorporating human factors frameworks for error classification (such as those in IEC 62366-2 guidance on usability engineering) in future studies would help pinpoint the exact nature of failures. Third, the measures of user experience in our study were limited. We relied on simple self-rating questions for task understanding and satisfaction, which, while informative, were not validated instruments. Using established usability questionnaires (e.g., the System Usability Scale or other multi-item scales) in future work would provide more reliable insights into how users perceive the ease or difficulty of the self-test. Additionally, our error data were based on observations by a single expert reviewer (with cross-checking by a second staff member), and we did not conduct a formal inter-rater reliability assessment. While coding was standardized by an experienced reviewer and cross-checked by the center head, the protocol did not include a formal inter-rater reliability (IRR) assessment, which constitutes a limitation. Future studies would benefit from having multiple independent raters and reporting inter-rater agreement statistics to strengthen confidence in the coding of errors. Finally, it should be acknowledged that we performed several exploratory subgroup analyses (e.g., breaking down error rates by subgroups within each test kit type and age strata) without statistical correction for multiple comparisons. These subgroup findings were used only for contextual understanding and were not the basis of our primary conclusions, but they should still be interpreted cautiously to avoid over-generalization.

Despite these limitations, the study provides clear evidence that older adults face disproportionate usability challenges when performing at-home COVID-19 self-testing, largely due to age-related cognitive factors. This has practical implications for public health and device design. At present, regulatory guidelines for medical device usability (including those by the U.S. FDA and other agencies) often require usability testing with a relatively small number of lay users (e.g., on the order of 15 users in formative or summative tests) [3, 12]. Our findings suggest that such minimal sample sizes may be insufficient to capture the full range of user capabilities and errors, especially for older populations. If a usability test happened to include few or no older participants, it could easily overlook the types of errors that we observed in this study. We therefore echo the call for more age-inclusive usability evaluations: test protocols should adequately represent older adults and, where feasible, include an additional usability test group of older participants to identify potential age-specific issues.

Moreover, our results reinforce the importance of applying “design for aging” principles in the development of home health products. Established human factors guidelines advocate designing systems that accommodate age-related sensory and cognitive changes by promoting simplicity, clarity, and flexibility in how information is conveyed. In the context of self-test kits, this could mean (in general terms) using more intuitive packaging, step-by-step visual guides, fail-safe features that catch mistakes, and other age-friendly design modifications – details of which we discuss in the Conclusion section. By interpreting our usability findings through the lens of gerontology and human factors knowledge, we highlight that the issue is not simply that “older people have more errors,” but rather that current self-test kit designs are not yet optimized for the cognitive and perceptual needs of older users. Addressing this gap will be crucial as we aim to make at-home diagnostics safe and effective for an aging population. Ultimately, improving the usability of medical self-tests for seniors not only benefits older individuals but also enhances overall public health resilience, ensuring that people of all ages can confidently and correctly use these important tools in managing their health.

## Conclusion

This study highlights the disproportionate usability challenges faced by older adults when using COVID-19 self-testing kits. Although overall usability was acceptable among younger participants, age consistently emerged as the strongest predictor of use errors, exceeding the effects of sex and education level. These errors were predominantly procedural, stemming from mismatches between task complexity and users’ cognitive abilities, rather than difficulties with manual execution. Based on observed error patterns, several design modifications may enhance safety and usability. First, instructions should prioritize step-by-step visuals and progressive disclosure, presenting only one required action at a time to minimize working-memory demands. Second, high-contrast pictograms, simplified typography, and clear labels for key components (e.g., buffer tube, filter cap, sample window) may help users with age-related visual decline. Third, components should provide strong tactile and visual feedback, such as audible clicks when caps are assembled or marked depth indicators for swab insertion, to prevent ambiguity during critical procedures. Fourth, dropper mechanisms should be engineered to automatically dispense the correct volume, reducing reliance on users’ interpretation of drop size or count. Finally, test devices could incorporate fail-safe cues—for example, a warning indicator when a required step is skipped or insufficient buffer volume is applied. Incorporating these age-inclusive, human-centered design elements would improve usability for older adults while maintaining ease of use for younger populations.

## Supplementary Information

Supplementary Material 1.

## Data Availability

The de-identified dataset and R code used for the analyses in this study have been deposited in the Zenodo repository and are publicly available under the DOI: https://doi.org/10.5281/zenodo.17273033.

## Abbreviations

ANSI/AAMI: American National Standards Institute/Association for the Advancement of Medical Instrumentation
COVID-19: Coronavirus disease 2019
CI: Confidence Interval
FDA: U.S. Food and Drug Administration
IEC: International Electrotechnical Commission
IRB: Institutional Review Board
IRR: Incidence Rate Ratio
KUTC: Korea University Guro Hospital Usability Test Center
MFDS: Ministry of Food and Drug Safety (Republic of Korea)
UFMEA: Use Failure Mode and Effects Analysis
OR: Odds Ratio
SD: Standard Deviation
