# Supplementary material for: Usability vulnerabilities of elderly adults in at-home COVID-19 self-test kits: findings from a South Korea usability study
Source: BMC Public Health. 2025 Nov 28;25:4361. doi: 10.1186/s12889-025-25798-z (PMC12750645; doi:10.1186/s12889-025-25798-z)
Supplement: Supplementary file 1 — Supplementary Material 1. [file 12889_2025_25798_MOESM1_ESM.docx]

Supplemental Material

**Supplementary Table S1. Usability test session configuration**

**
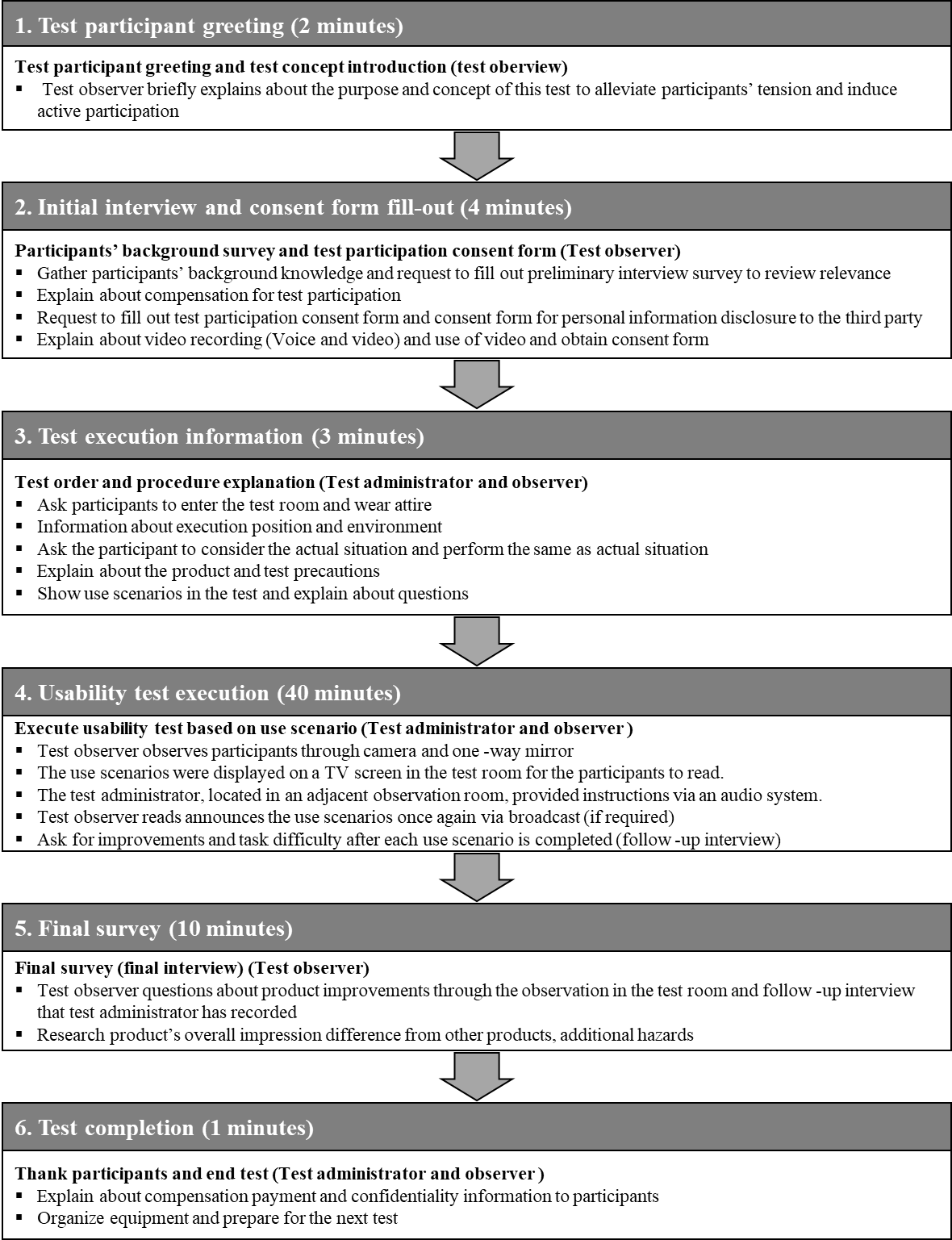
**

**Supplementary Table S2. Usability test participant flow diagram**

**
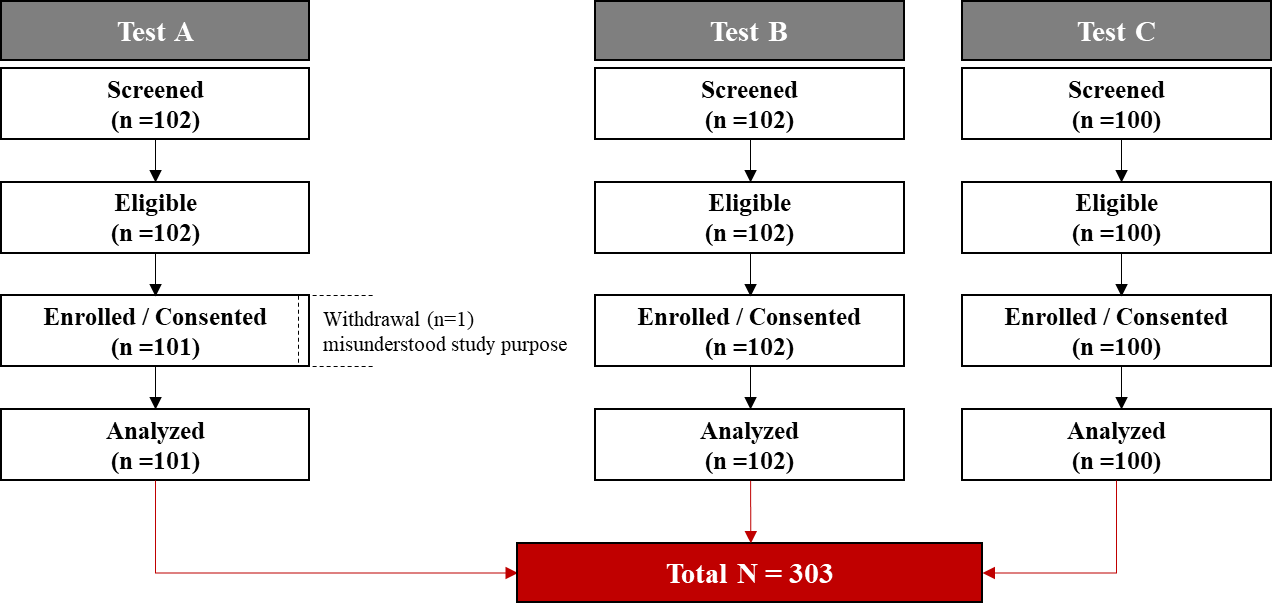
**

**Supplementary Table S3. Task classification**

| Standardized Task No. | Standardized Task Name | Test A: Manufacturer-Defined Steps | Test B: Manufacturer-Defined Steps | Test C: Manufacturer-Defined Steps |
| --- | --- | --- | --- | --- |
| 1 | Check expiration date and storage conditions | 1. Check expiration date 2. Check storage conditions and temperature | 1. Check expiration date 2. Check storage conditions and temperature | 1. Check validity period |
| 2 | Prepare for test and sanitize hands | 3. Prepare test area 4. Sanitize hands 5. Understand sanitization instructions | 3. Sanitize hands | 2. Sanitize hands |
| 3 | Check and understand kit components | 6. Check components 7. Understand precautions when checking components 8. Understand components | 4. Check components | 3. Check components |
| 4 | Prepare buffer tube for sample | 9. Remove foreign substances from nose 10. Prepare gloves and timer 11. Prepare tube 12. Mount tube | 5. Remove cap from solution tube 6. Place solution tube in holder | 4. Remove film from extraction reagent 5. Place extraction reagent in holder |
| 5 | Prepare swab for sample collection | 13. Prepare swab | 7. Prepare swab | 6. Prepare sterile swab |
| 6 | Collect nasal sample | 14. Collect sample 15. Understand collection from both nostrils 16. Understand collection site | 8. Collect sample | 7. Collect sample |
| 7 | Mix sample with buffer solution | 17. Extract sample 18. Break swab | 9. Mix solution | 8. Extract sample 9. Dispose of sterile swab |
| 8 | Prepare test device and assemble filter cap | 19. Close filter cap 20. Prepare test device | 10. Check desiccant color 11. Prepare test device | 10. Close filter cap |
| 9 | Dispense sample mixture into test device | 21. Mix sample 22. Understand drop location 23. Dispense drops | 12. Instill solution | 11. Dispense drops |
| 10 | Wait for and read the results | 24. Understand time to result | 13. Understand time to result | 12. Understand time to result |

**Supplementary Table S4. Pooled models adjusted for kit and calendar time.**

| **Model** | **N** | **AIC** | **BIC** | **Estimate** | **SE** | **OR/IRR** | **95% CI (OR/IRR)** | **p-value** |
| --- | --- | --- | --- | --- | --- | --- | --- | --- |
| Logit + Kit FE, cluster-SE by Kit | 303 | 229.4654 | 251.7478 | 0.090716 | 0.005976 | 1.094958 | [1.082, 1.108] | <0.001 |
| Poisson + Kit FE, cluster-SE by Kit (IRR) | 303 | 479.8163 | 502.0987 | 0.081246 | 0.007822 | 1.084637 | [1.068, 1.101] | <0.001 |
| NegBin + Kit FE, cluster-SE by Kit (IRR) | 303 | 399.4235 | 425.4196 | 0.071106 | 0.002641 | 1.073695 | [1.068, 1.079] | <0.001 |
| Logit Mixed: (1\|Kit) | 303 | 229.8255 | 248.3941 | 1.094688 | 0.016137 | 1.094688 | [1.064, 1.127] | <0.001 |
| Poisson Mixed: (1\|Kit) (IRR) | 303 | 485.6467 | 504.2154 | 1.086945 | 0.009192 | 1.086945 | [1.069, 1.105] | <0.001 |
| NegBin Mixed: (1\|Kit) (IRR) | 303 | 401.1679 | 423.4503 | 1.079001 | 0.013434 | 1.079001 | [1.053, 1.106] | <0.001 |
| Notes & Footnotes  Pooled models adjusted for kit (Test A/B/C) and calendar time (days since 2021-06-01).  Mixed-effects models include a random intercept for kit; fixed-effects specifications reported as sensitivity.  OR (any use error) and IRR (error count) are per 1-year increase in age.  Exact p-values reported to three decimals; values <0.001 shown as '<0.001'. | | | | | | | | |

**Supplementary Table S5. Model diagnostics and selection rationale.**

| **Outcome** | **Model specification** | **Dispersion ratio (Poisson)** | **ZI p (Poisson RE)** | **ZI p (NB RE)** | **Decision / Interpretation** |
| --- | --- | --- | --- | --- | --- |
| Error count | Poisson Mixed: (1\|Kit) | 3.659 | 0.04 |  | NB preferred (dispersion>1.2) |
| Error count | NegBin Mixed: (1\|Kit) |  |  | 0.964 | Zero inflation not indicated under NB; ZINB not needed |
| Notes & Footnotes  Dispersion ratio > 1.2 indicates overdispersion favoring negative binomial over Poisson.  DHARMa zero-inflation tests: non-significant p-values indicate no material zero-inflation. | | | | | |
